# Evolutionary diversity of the control of the azole response by Tra1 across yeast species

**DOI:** 10.1093/g3journal/jkad250

**Published:** 2023-10-27

**Authors:** Gabriela Marsiglio Nunes Librais, Yuwei Jiang, Iqra Razzaq, Christopher J Brandl, Rebecca S Shapiro, Patrick Lajoie

**Affiliations:** Department of Anatomy and Cell Biology, The University of Western Ontario, London, Ontario, N6A 5C1, Canada; Department of Anatomy and Cell Biology, The University of Western Ontario, London, Ontario, N6A 5C1, Canada; Department of Molecular and Cellular Biology, University of Guelph, Guelph, Ontario N1G 2W1, Canada; Department of Biochemistry, The University of Western Ontario, London, Ontario, N6A 5C1, Canada; Department of Molecular and Cellular Biology, University of Guelph, Guelph, Ontario N1G 2W1, Canada; Department of Anatomy and Cell Biology, The University of Western Ontario, London, Ontario, N6A 5C1, Canada

**Keywords:** Tra1, SAGA complex, azole resistance, fungal pathogen, ergosterol, *Candida albicans*, *CDR1*

## Abstract

Tra1 is an essential coactivator protein of the yeast SAGA and NuA4 acetyltransferase complexes that regulate gene expression through multiple mechanisms including the acetylation of histone proteins. Tra1 is a pseudokinase of the PIKK family characterized by a C-terminal PI3K domain with no known kinase activity. However, mutations of specific arginine residues to glutamine in the PI3K domains (an allele termed *tra1_Q3_*) result in reduced growth and increased sensitivity to multiple stresses. In the opportunistic fungal pathogen *Candida albicans*, the *tra1_Q3_* allele reduces pathogenicity and increases sensitivity to the echinocandin antifungal drug caspofungin, which disrupts the fungal cell wall. Here, we found that compromised Tra1 function, in contrast to what is seen with caspofungin, increases tolerance to the azole class of antifungal drugs, which inhibits ergosterol synthesis. In *C. albicans*, *tra1_Q3_* increases the expression of genes linked to azole resistance, such as *ERG11* and *CDR1*. *CDR1* encodes a multidrug ABC transporter associated with efflux of multiple xenobiotics, including azoles. Consequently, cells carrying *tra1_Q3_* show reduced intracellular accumulation of fluconazole. In contrast, a *tra1_Q3_ Saccharomyces cerevisiae* strain displayed opposite phenotypes: decreased tolerance to azole, decreased expression of the efflux pump *PDR5*, and increased intracellular accumulation of fluconazole. Therefore, our data provide evidence that Tra1 differentially regulates the antifungal response across yeast species.

## Introduction

Fungal infections represent a major public health concern with over a billion infections each year resulting in more than 1.5 million deaths (Bongomin *et al.* 2017). Members of the *Candida* genus, including *Candida albicans*, are opportunistic pathogens that can cause a wide range of severe infections in susceptible populations, such as the elderly or immunocompromised individuals (Pappas *et al.* 2018). *Candida* species represent the leading cause of fungal infection–related deaths worldwide (Rokas 2022). Treatment of *Candida* infection (candidiasis) is unfortunately limited to 4 major classes of antifungal drugs: echinocandins, azoles, polyenes, and flucytosines. Echinocandins, such as caspofungin, target the fungal cell wall by inhibiting the synthesis of the carbohydrate β-1,3-glucan (Balashov *et al.* 2006; Lee *et al.* 2012), while azoles, such as fluconazole and miconazole, inhibit the synthesis of ergosterol, thereby compromising the lipid composition of fungal membranes (Kuznetsov 2021). Azoles specifically inhibit the 14α-demethylase, encoded by the *C. albicans ERG11* gene, in the ergosterol synthesis pathway. Consequently, mutations in *ERG11* are one of the main sources of acquired azole resistance (Wu *et al.* 2017; Kaur and Nobile 2022).

Tra1 is an essential component of the SAGA and NuA4 complexes that regulate the acetylation of both histone and nonhistone substrates (Grant *et al.* 1997; Clarke *et al.* 1999; Steunou *et al.* 2014; Downey 2021) in all eukaryotic cells, including fungi (Saleh *et al.* 1998; Grant *et al.* 1998; Allard *et al.* 1999). Recently, Tra1 was shown to be essential for viability in *C. albicans* (Razzaq *et al*. 2021; Rashid *et al*. 2022). Tra1 is essential due to its function in NuA4. This is highlighted in the fission yeast *Schizosaccharomyces pombe* where the SAGA-incorporated Tra1 is dispensable for viability while conversely, the Nua4-localized Tra2 is essential (Helmlinger *et al.* 2011). Many components of the SAGA and NuA4 complexes regulate different aspects of the *C. albicans* antifungal response and pathogenicity (Laprade *et al.* 2002; Lu *et al.* 2008; Sellam *et al.* 2009; Askew *et al.* 2009; Chang *et al.* 2015; Shivarathri *et al.* 2019; Razzaq *et al.* 2021; Rashid *et al.* 2022). However, the functions of Tra1 in *C. albicans* remain poorly understood.

Tra1 is a member of the phosphoinositide 3-kinase-related kinase (PIKK) family, but unlike other family members such as Tor1, it does not possess any detectable kinase activity due to the lack of specific kinase motifs (McMahon *et al.* 1998; Saleh *et al.* 1998; Helmlinger *et al.* 2011). Despite its pseudokinase nature, we have previously shown that mutation of key arginine residues to glutamine in the Tra1 PI3K domain, an allele termed *tra1_Q3_*, is associated with increased sensitivity to various stress conditions, including cell wall stress, protein misfolding, and high temperature in the budding yeast *Saccharomyces cerevisiae* (Berg *et al*. 2018). *tra1_Q3_* mutants also display increased sensitivity to acid stress, impaired growth in respiratory conditions, and reduced chronological lifespan (Bari *et al.* 2022). Similar to their *S. cerevisiae* counterparts, *C. albicans tra1_Q3_* mutants display increased sensitivity to cell wall stress induced by caspofungin, as well as reduced biofilm formation and pathogenicity (Razzaq *et al.* 2021). However, the impact of Tra1 on *C. albicans* resistance to other classes of antifungal drugs remains unknown. Hence, here we tested the effect of the *tra1_Q3_* mutation to modulate sensitivity to other antifungal compounds.

## Materials and methods

### Reagents

Fluconazole, amphotericin B, miconazole, FK506, rhodamine 6G (R6G), and caspofungin were from MilliporeSigma. Propidium iodide (PI) was from Thermo Fisher Scientific.

### Yeast strains and growth conditions

The *tra1* mutant strains used in this study were previously described (Berg *et al.* 2018; Razzaq *et al.* 2021) and are listed in Supplementary Table 1. Both *S. cerevisiae* and *C. albicans* strains were cultured in YPD (2% Bacto peptone, 1% yeast extract, and 2% glucose) unless noted. Cells were grown in liquid YPD overnight at 30°C with shaking. The next day, cells were diluted at 1:10 ratio and then incubated for 2 h at 30°C with shaking. Cell growth on agar plates was measured as previously described (Petropavlovskiy *et al.* 2020).

### Fluorescent fluconazole probe uptake

The fluorescent fluconazole probe (RB510) was added to log phase cells cultured at 30°C with shaking to a final concentration of 1 µg/mL as previously described (Benhamou *et al.* 2017). Next, the cells were incubated in the dark for 60 min at 30°C with shaking. Then, cells were washed with phosphate-buffered saline (PBS). The mean fluorescence intensity of the probe was measured with a BD FacsCelesta flow cytometer. Data were collected from 30,000 cells per time point using a 561-nm yellow–green laser. Mean fluorescent intensity was calculated using FlowJo. No gates were applied.

### Cell viability assay

*Saccharomyces cerevisiae* cells were grown in liquid SC media lacking leucine overnight at 30°C with shaking. The next day, cells were diluted at 1:5 ratio and then incubated for 5 h at 30°C with shaking until the log phase in 50-mL culture flasks. Next, cells were equalized to OD_600_ = 0.80, treated or not with 20 µg/mL of fluconazole, and incubated with shaking for 1–24 h at 30°C. After each time point, 1 mL of cells was pelleted by centrifugation and resuspended in a final volume of 1000 µL PBS. For positive control, 1 sample of each strain was boiled for 15 min at 100°C. Five hundred microliters of cell suspension was stained with 2.5 µL of 1 mg/mL PI solution, and cells were incubated in the dark for 10 min at room temperature as previously described (Chadwick *et al.* 2016). Data were collected from 30,000 cells per time point using a BD FACSCelesta flow cytometer (BD Biosciences) equipped with a 561-nm yellow–green laser. Analysis was performed using FlowJo.

### Gene expression analysis

Total RNA was isolated using the MasterPure Yeast RNA purification kit (Lucigen) according to the manufacturer’s instructions. cDNA was synthesized from 2.5 µg total RNA using the SuperScript IV VILO Master Mix (Thermo Fisher Scientific) according to the manufacturer’s instructions. qRT-PCR for *ERG11* and *CDR1/PDR5* together with *ACT1/TDH3* as housekeeping gene was amplified from the synthesized cDNA using primers listed in Supplementary Table 2 with a QuantStudio 3 real-time PCR system using the ΔΔCT method (Thermo Fisher Scientific).

### Efflux of R6G

To measure *C. albicans* drug efflux capacity, R6G efflux was measured by fluorescence assay with whole cells. *Candida albicans* were grown in liquid YPD overnight at 30°C with shaking in 10-mL culture tubes. First, cells were diluted at a 1:10 ratio and then incubated for 2 h at 30°C with shaking until the log phase. Next, cells were pelleted by centrifugation, washed with 5 mL PBS (pH 7), resuspended in 2 mL PBS, and incubated for 1 h at 30°C with shaking in PBS to energy-deprived cells. R6G was added at a concentration of 4 µM, and the incubation continued for 1 h, thus facilitating R6G accumulation. After this incubation, cells were sedimented by centrifugation, washed with PBS, and resuspended in a final volume of 200 µL PBS. Fifty microliters of individual strains was diluted in 150 µL PBS and aliquoted in a 96-well microtiter plate, which was placed in a BioTek Cytation5 Cell Imaging Multimode Reader (Agilent) with temperature control set at 30°C. Baseline emission of fluorescence (excitation wavelength: 584 nm; emission wavelength: 625 nm) and OD_600_ was recorded for 0, 2, and 4 min. Glucose (2% final concentration) was next added to each strain to initiate R6G efflux. As a negative control, no glucose was added to separate aliquots of each strain. Data points were recorded in triplicate for 60 min at 2-min intervals. Data were plotted as the ratio of fluorescence value/OD_600_ data point.

## Results and discussion

### Compromise of Tra1 function differentially impacts azole resistance across yeast species

**Fig. 1.**
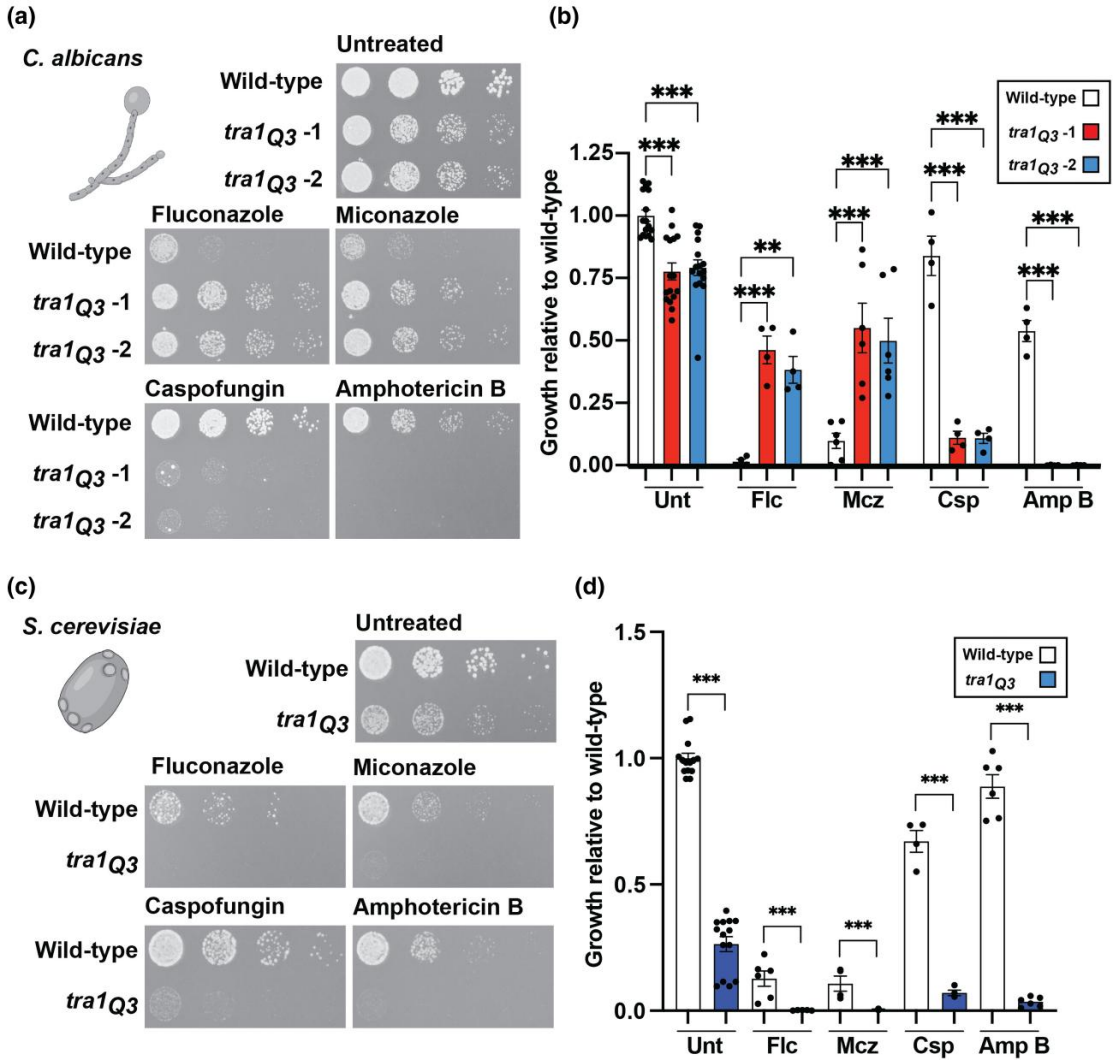
*tra1_Q3_* results in differential resistance to azoles across yeast species. a) Wild-type and *tra1_Q3_ C. albicans* cells were spotted on YPD agar plates at 30°C without treatment or containing either 20 µg/mL fluconazole (Flc), 0.5 µg/mL miconazole (Mcz), 0.05 µg/mL caspofungin (Csp), or 0.5 µg/mL amphotericin B (Amp B). b) Quantification of the growth relative to untreated wild type is shown in the bar graph. The second dilution was used for quantification. Data are presented ± SEM *n* ≥ 4 and ***P < 0.0003; **P < 0.01. c) Wild-type and *tra1_Q3_ S. cerevisiae* cells were spotted on YPD agar plates at 30°C without treatment or containing either 20 µg/mL Flc, 0.3 µg/mL Mcz, 0.10 µg/mL Csp, or 0.5 µg/mL Amp B. d) Quantification of the growth relative to untreated wild type is shown in the bar graph. Second dilution was used for quantification. Data are presented ± SEM *n* ≥ 4 and ***P < 0.0003. Yeast illustrations were generated using biorender.com.

To test the effect of the *tra1_Q3_* allele on the *C. albicans* antifungal resistance, wild-type cells and cells carrying the mutations were spotted on agar plates containing the echinocandin caspofungin, the azoles miconazole and fluconazole, or amphotericin B (Fig. 1a and b). While caspofungin disrupts the fungal cell wall by inhibiting the β-(1,3)-D-glucan synthase, both azoles and amphotericin B affect fungal membranes. Azoles inhibit ergosterol synthesis by inhibiting the cytochrome P450 enzyme 14α-demethylase (Hitchcock 1991; Wu *et al.* 2017). Polyenes, such as amphotericin B, disrupt membrane integrity by directly binding to ergosterol (Nett *et al.* 2010). As previously observed (Razzaq *et al.* 2021), 2 independently generated *tra1_Q3_* mutants display increased sensitivity to caspofungin. Similarly, we observed increased sensitivity to amphotericin B. Unexpectedly, *tra1_Q3_* cells showed increased tolerance to the azoles miconazole and fluconazole compared to wild type. While both azoles and amphotericin B affect fungal membrane integrity, they do so via very distinct mechanisms; mechanisms for the development of antifungal resistance to these drugs are also very different (Lee *et al.* 2020). Interestingly, deleting other SAGA complex components such as *SPT7*, *SPT8*, and *ADA2* sensitizes *C. albicans* to both caspofungin and fluconazole (Bruno *et al.* 2006; Sellam *et al.* 2009; Rashid *et al.* 2022), whereas deleting other components, such as *NGG1* and *UPB8*, has no effect on antifungal drug resistance (Rashid *et al.* 2022). Therefore, different SAGA components and/or modules may differentially contribute to antifungal drug resistance. Moreover, due to its incorporation in both SAGA and NuA4, Tra1 has additional roles that may contribute to the phenotypes observed here. Indeed, while the role for NuA4 in hyphal growth has been characterized (Lu *et al.* 2008), how it regulates antifungal drug resistance remains unclear.

In contrast, in *S. cerevisiae*, *tra1_Q3_* cells are hypersensitive to caspofungin, amphotericin B, and azoles (Fig. 1c and d). This is consistent with previous results showing that loss of Tra1 function in budding yeast sensitizes cells to multiple stresses such as heat shock, protein misfolding, ageing, cell wall perturbation, and DNA damage (Mutiu *et al.* 2007; Hoke *et al.* 2008, 2010; Berg *et al.* 2018; Cheung and Díaz-Santín 2019; Jiang *et al.* 2019; Bari *et al.* 2022). The differential sensitivity to azoles between *C. albicans* and *S. cerevisiae* suggests that differences exist between the genes impacted by Tra1 across yeast species.

### Tra1 regulates the expression of genes associated with azole resistance

**Fig. 2.**
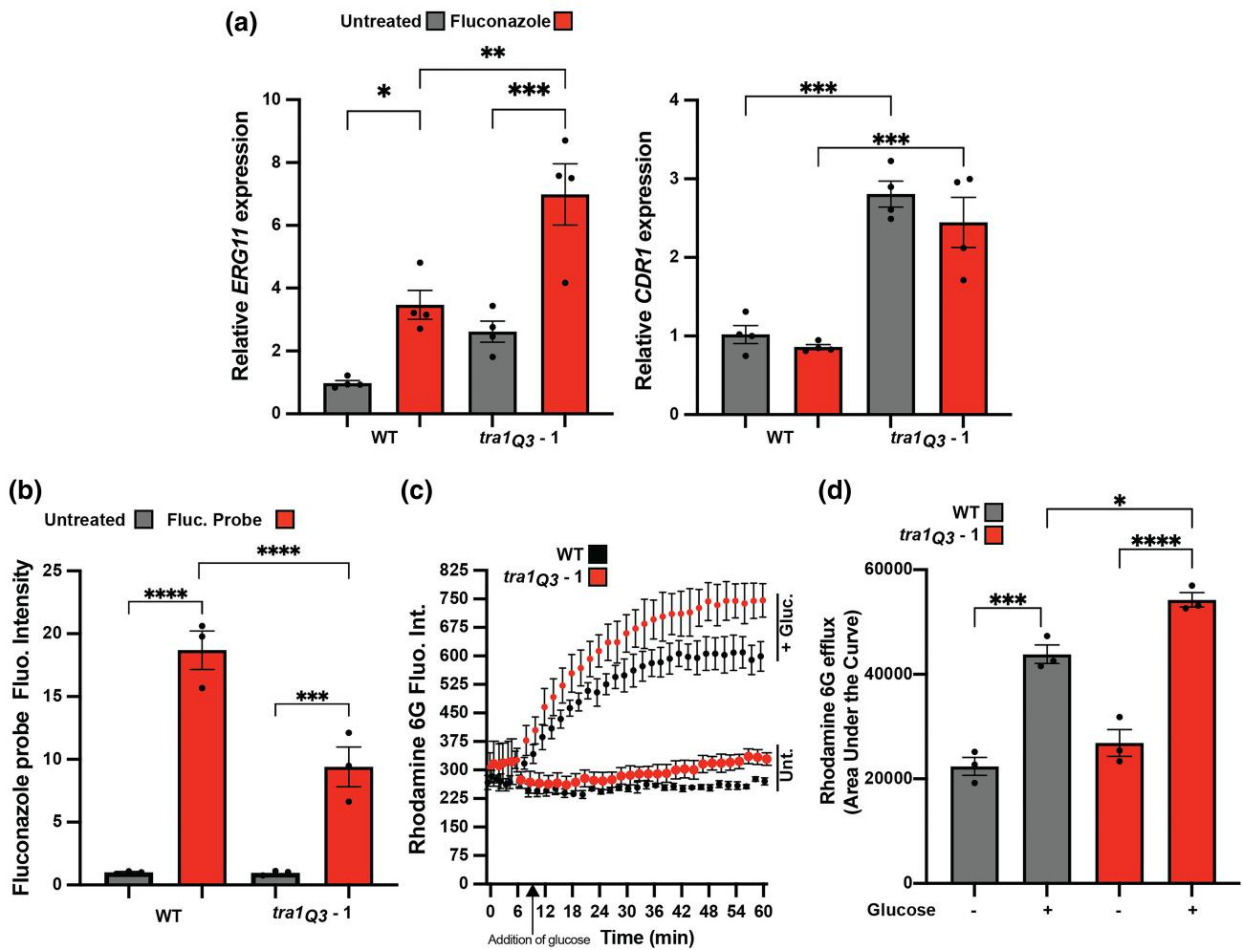
*Candida albicans tra1_Q3_* mutants display phenotypes associated with azole resistance. a) *tra1_Q3_* cells show increased expression of genes linked to azole resistance. Wild-type and *tra1_Q3_ C. albicans* cells were incubated with 20 µg/mL fluconazole for 1 h and the expression of *ERG11* and *CDR1* was assessed by qRT-PCR. *n* = 4. b) *tra1_Q3_* cells show reduced accumulation of intracellular fluconazole. Wild-type and *tra1_Q3_ C. albicans* cells were incubated with a fluorescent fluconazole probe for 1 h, and mean fluorescent intensity of intracellular fluconazole was assessed by flow cytometry. *n* = 3. c) R6G efflux is increased in *tra1_Q3_* cells. Energy-depleted cells were incubated with R6G for 1 h and then left untreated or treated with glucose to induce efflux. Mean rhodamine 6D fluorescence was monitored over time. *n* = 3. d) Quantification of the area under the curve calculated from R6G release assays is shown in the bar graph. *n* = 3. Means are shown ± SEM ****P < 0.0001; ***P < 0.0003; **P < 0.01; *P < 0.05.

Given the decreased azole susceptibility in *tra1_Q3_* cells, we next assessed the expression of genes previously associated with azole resistance in *C. albicans*. Specifically, we addressed the expression of *ERG11* and *CDR1*. Mutations in *ERG11* resulting in overexpression or loss of drug affinity are associated with azole resistance in multiple *C. albicans* clinical isolates (Franz *et al.* 1998; MacPherson *et al.* 2005; Flowers *et al.* 2015). Cdr1 is a member of the ATP-binding cassette transporter family associated with multidrug resistance (Dogra *et al.* 1999; Holmes *et al.* 2008). Cdr1 is known for its role in fluconazole efflux and acquired multidrug resistance in clinical isolates of *C. albicans* (Holmes *et al*. 2008). As shown in Fig. 2a, there was a significant increase in the expression of *CDR1* and *ERG11* in *tra1_Q3_* cells relative to wild type both in untreated conditions (both 2.8-fold) and upon the addition of fluconazole (2.7- and 2.1-fold, respectively). In the past, we have shown, in budding yeast, that components of the SAGA complex can act as repressors of transcription under different conditions (Ricci *et al.* 2002).

In light of the increased expression of *CDR1* in *tra1_Q3_* cells in *C. albicans*, we investigated whether compromising Tra1 function affects drug efflux and intracellular bioavailability. To do so, we took advantage of a fluorescent probe that allows the real-time imaging of azole uptake in fungal cells (Benhamou *et al.* 2017). We found that intracellular accumulation of fluorescently tagged azole is significantly reduced by 50% in *tra1_Q3_* cells (Fig. 2b). Since *CDR1* expression has been linked to increased efflux of fluconazole in fungi (Hernáez *et al.* 1998; Kim *et al.* 2019), we tested whether *tra1_Q3_ C. albicans* have higher drug efflux capacity using the well-characterized Cdr1 efflux substrate R6G (Maesaki *et al.* 1999). Our findings support that there is increased efflux of R6G in *tra1_Q3_* cells (Fig. 2c and d), suggesting that elevated expression of *CDR1* contributes to greater azole tolerance. This upregulation of efflux pumps may explain the unique azole resistance of *tra1_Q3_*. Upregulation of efflux pumps is a well-established mechanism of azole resistance but has not been linked to resistance of amphotericin B or caspofungin (Lee *et al.* 2020).

In response to azole, *C. albicans* activates the calcineurin pathway, which is essential for virulence (Blankenship *et al.* 2003; Juvvadi *et al.* 2014). Inhibiting calcineurin-dependent signaling with FK506 increases susceptibility to azole in *C. albicans* (Cruz *et al*. 2002; Uppuluri *et al*. 2008; Khodavaisy *et al*. 2023). Thus, we tested whether FK506 alleviates the azole resistance observed in *tra1_Q3_* cells. Indeed, we found that azole tolerance in *tra1_Q3_* cells is suppressed by treatment with the calcineurin inhibitor (Supplementary Fig. 1). Together, our findings suggest that while some cellular mechanisms associated with azole resistance, such as *ERG11* and *CDR1* expression, are increased in *tra1_Q3_* cells, drug tolerance still requires a functional calcineurin pathway. This is in agreement with previous studies showing that the expression of *CDR1* and ergosterol biosynthesis genes is independent of calcineurin signaling (Cruz *et al.* 2002; Jia *et al.* 2016).

**Fig. 3.**
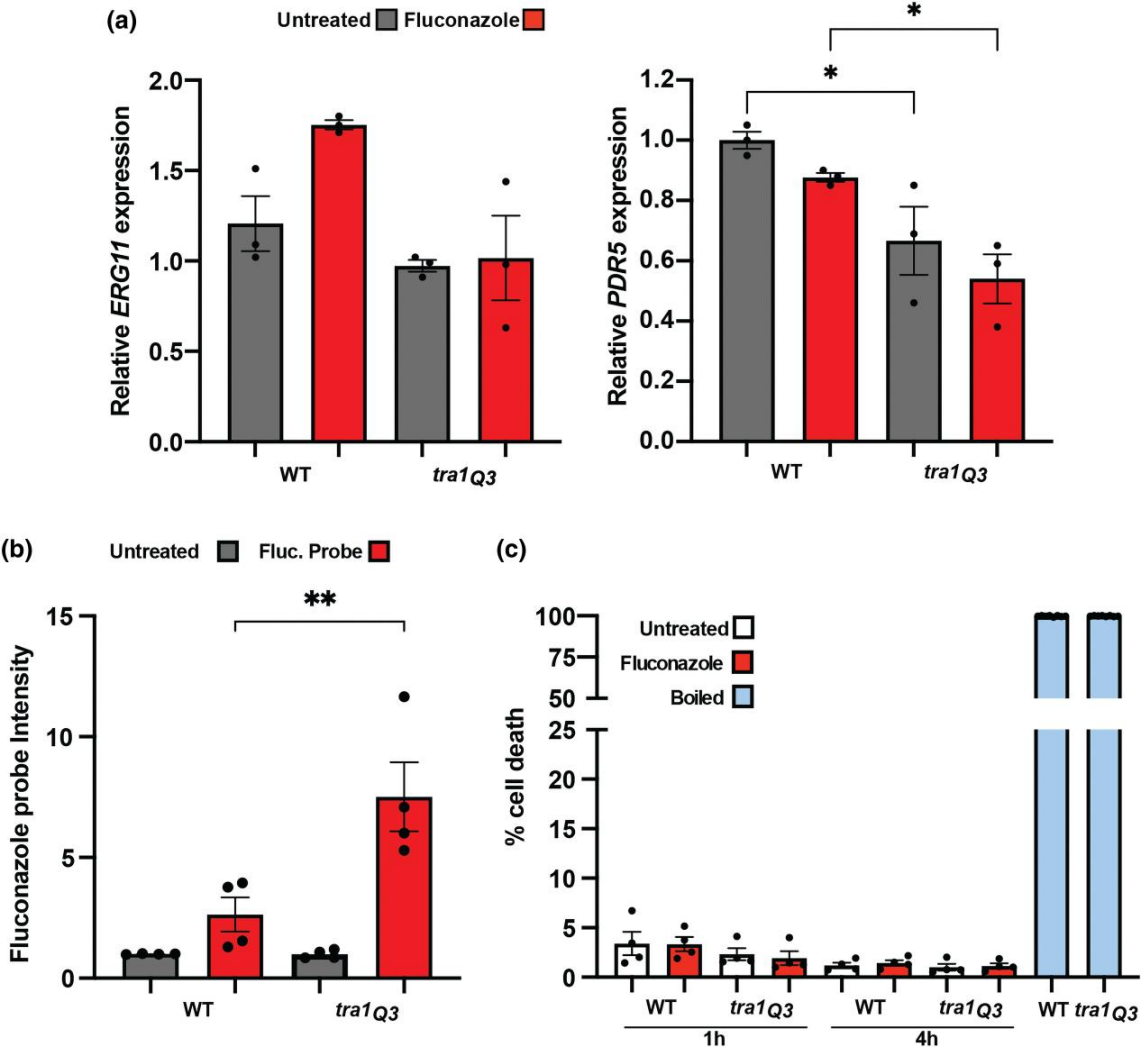
*Saccharomyces cerevisiae tra1_Q3_* mutants display phenotypes associated with increased azole sensitivity. a) *tra1_Q3_* cells show decreased expression of genes linked to azole resistance. Wild-type and *tra1_Q3_ C. albicans* cells were incubated with 20 µg/mL fluconazole for 1 h and the expression of *ERG11* and *PDR5* was assessed by qRT-PCR. *n* = 3. b) *tra1_Q3_* cells show increased accumulation of intracellular fluconazole. Wild-type and *tra1_Q3_* cells were incubated with a fluorescent fluconazole probe for 1 h, and mean fluorescent intensity of intracellular fluconazole was assessed by flow cytometry. *n* = 4. c) *tra1_Q3_* cells do not show increased cell death upon fluconazole treatment. Wild-type and *tra1_Q3_ S. cerevisiae* cells were incubated with 20 µg/mL fluconazole for 1 and 4 h and stained with PI to assess cell viability. Boiled cells are shown as positive control. The percentage of PI-positive cells was assessed by flow cytometry and presented in a bar graph. *n* = 4. All data are shown ± SEM *P < 0.05 **P<0.01.

Next, we investigated the effect of *tra1_Q3_* on azole resistance mechanisms in budding yeast given the differences between *C. albicans* and *S. cerevisiae* (Fig. 1). In *S. cerevisiae*, SAGA regulates gene expression in response to changes in sterol content (Dewhurst-Maridor *et al.* 2017). NuA4 is also linked to sterol metabolism, and *eaf1Δ* cells display increased accumulation of ergosterol esters (Pham *et al.* 2022). Unlike *C. albicans*, *S. cerevisiae tra1_Q3_* cells did not demonstrate significant changes in *ERG11* expression, as compared to the wild type (Fig. 3a). This difference with our *C. albicans* data is consistent with a potential rewiring of the role of Tra1 in the transcription of sterol genes between species. Indeed, rewiring of SAGA functions between *S. cerevisiae* and *C. albicans* has been previously reported. Spt3 negatively regulates filamentous growth (a key virulence trait) in *C. albicans* but has an opposite role in *S. cerevisiae* (Laprade *et al.* 2002). Moreover, Sch9 has been shown to play a critical role in chromosome segregation in *C. albicans*, a function absent in budding yeast (Varshney *et al.* 2015). Functional rewiring between the 2 species is not surprising given that the phylogenetic distance between *S. cerevisiae* and *C. albicans* is approximately the same as between humans and sponges (Shen *et al.* 2018).

Since the *tra1_Q3_* cells showed increased expression of *CDR1* in *C. albicans*, we investigated its impact on the expression of the *PDR5* ABC transporter (the *CDR1* homolog) in *S. cerevisiae*. We found reduced expression of *PDR5* in *tra1_Q3_* cells (Fig. 3a). This is consistent with the role of SAGA in the regulation of *PDR5* expression (Gao *et al.* 2004) and reduced global transcription of SAGA-regulated genes previously observed in the *tra1_Q3_* mutant (Berg *et al.* 2018). Consistent with reduced *PDR5* expression, intracellular accumulation of fluorescently labeled fluconazole increased in *tra1_Q3_* cells (Fig. 3b).

In contrast to other antifungal drugs, such as amphotericin B, which behave in a fungicidal manner, azoles are fungistatic, thus inducing minimal cell death in multiple yeast species (Manavathu *et al.* 1998). For this reason, we investigated whether the decreased growth observed in the *tra1_Q3_ S. cerevisiae* cells treated with fluconazole was associated with changes in cell death. Wild-type and *tra1_Q3_ S. cerevisiae* cells were treated with fluconazole and stained with PI at different time intervals for up to 24 h (Fig. 3c). No significant differences were observed, indicating that the effect of fluconazole remains fungistatic within the *tra1_Q3_* cells. Finally, treatment of *S. cerevisiae* with the calcineurin inhibitor FK506 sensitized cells to fluconazole (Supplementary Fig. 2a). While *tra1_Q3_* cells were not inherently sensitive to the inhibitor, the cells displayed a synthetic negative interaction when crossed with a *cnb1Δ* mutant (Supplementary Fig. 2b), which encodes a calcineurin subunit (Cyert and Thorner 1992). We previously observed a similar phenotype with a distinct Tra1 mutant (Hoke *et al.* 2008). These results suggest that Tra1 and calcineurin, like in *C. albicans*, function within distinct signaling pathways.

### Conclusions and perspectives

While biochemical and structural studies have extensively characterized Tra1 functions in model yeast, its specific impact for gene expression in fungal pathogens such as *C. albicans* is poorly understood. How compromise of Tra1 function leads to the upregulation of genes linked to azole resistance will require more detailed investigations to define genome-wide occupancy of coactivator complexes and their role in activation, repression, and maintenance of transcription under different conditions.

**Fig. 4.**
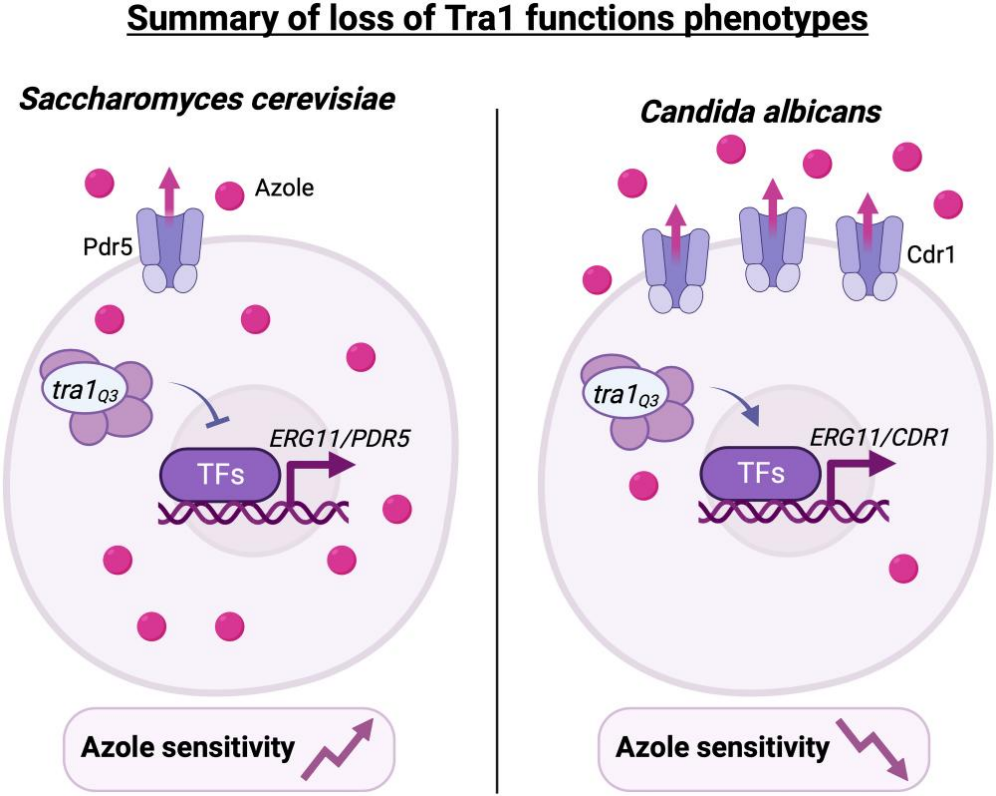
Summary of *tra1_Q3_* phenotypes associated with azole treatment in *S. cerevisiae* and *C. albicans*. In *S. cerevisiae*, compromise of Tra1 function associated with the *tra1_Q3_* allele results in decreased expression of genes associated with azole resistance such as *ERG11* and *PDR5*. Consequently, *tra1_Q3_* cells show increased accumulation of intracellular azole. In *C. albicans*, *tra1_Q3_* is linked to the increased expression of *ERG11* and *CDR1*, increased efflux of azole, and consequently, increased drug resistance. Created with Biorender.com.

Here, we also show that negatively impacting Tra1 function has opposite effects with regard to azole tolerance between *S. cerevisiae* and *C. albicans* (Fig. 4). This highlights the evolutionary diversity of the control of the antifungal response by Tra1-containing complexes. Future investigations should aim at defining the breadth of Tra1 functions across fungi. *Nakaseomyces* (*Candida*) *glabrata* is the second most common cause of candidiasis (McCarty *et al.* 2021) but is evolutionarily more closely related to *S. cerevisiae* than other pathogenic *Candida* species. *Nakaseomyces glabrata* is highly dependent upon the upregulation of *CDR1* and *CDR2* in response to antifungal stress (Sanglard *et al.* 2009) and thus could serve as a compelling comparison to assess this divergent role of Tra1 among yeast species. Finally, similar to other members of the PIKK family, such as Tor, Tra1 should be druggable. However, our data suggest that its potential as a candidate for combinational therapy with antifungal drugs would have to be considered carefully.

## Supplementary Material

jkad250_Supplementary_Data

## Data Availability

Strains and plasmids are available upon request. The authors affirm that all data necessary for confirming the conclusions of the article are present within the article, figures, and tables. Supplemental material available at G3 online.
