# Supplementary material for: Evolutionary diversity of the control of the azole response by Tra1 across yeast species
Source: G3 (Bethesda). 2023 Oct 27;14(2):jkad250. doi: 10.1093/g3journal/jkad250 (PMC10849324; doi:10.1093/g3journal/jkad250)
Supplement: jkad250_Supplementary_Data [file jkad250_supplementary_data.zip › G3-2023-404639-T_Supplemental_Figure_Legends.docx]

**Figure S1: Treatment with FK506 alleviates the fluconazole resistance of *C. albicans tra1_Q3_* cells.** Wild-type and *tra1_Q3_* *C. albicans* cells were spotted on YPD agar plates without treatment or containing either 20 µg/mL fluconazole, 2 µg/ml FK506 or a combination of both.

**Figure S2: *S. cerevisiae* *tra1_Q3_* shows synthetic negative genetic interaction with *CNB1*. (A)** Wild-type, *cnb1Δ*, *tra1_Q3_* and the double mutant *tra1_Q3_* were spotted on YPD agar plates without treatment or containing either 20 µg/mL fluconazole, 2 µg/ml FK506 or a combination of both. **(B)** Wild-type, *cnb1Δ*, *tra1_Q3_* and the double mutant were grown in YPD and OD_600_ was measured over time and growth curves were generated. For each strain the area under the curve was calculated and shown in insert.
