# Supplementary material for: Evolutionary diversity of the control of the azole response by Tra1 across yeast species
Source: G3 (Bethesda). 2023 Oct 27;14(2):jkad250. doi: 10.1093/g3journal/jkad250 (PMC10849324; doi:10.1093/g3journal/jkad250)
Supplement: jkad250_Supplementary_Data [file jkad250_supplementary_data.zip › G3-2023-404639-T_Table_S1.docx]

**Table S1: Strains used in this study**

| **Yeast Species** | **Name** | **Genotype** | **Reference** |
| --- | --- | --- | --- |
| *Candida albicans* | fRS302 | NAT-Cas9 | [(Razzaq *et al.* 2021)](https://paperpile.com/c/VKY8u6/k8LS) |
|  | fRS318  (*tra1_Q3_*-1) | NAT-Cas9 tra1Q3/tra1Q3  (K3471Q, R3472Q,  R3538Q) |  |
|  | fRS319  (*tra1_Q3_*-2) | NAT-Cas9 tra1Q3/tra1Q3  (K3471Q, R3472Q,  R3538Q) |  |
| *Saccharomyces cerevisiae* | CY4353 | MATα his3Δ0 leu2Δ0  ura3Δ0 TRA1-HIS3 +DED1pr-YHR100-LEU2 | [(Hoke *et al.* 2010)](https://paperpile.com/c/VKY8u6/CO1V) |
|  | CY6582 | MATα his3Δ0 leu2Δ0 ura3 Δ0 *tra1_Q3_*-HIS3  +DED1pr-YHR100-LEU2 | [(Berg *et al.* 2018)](https://paperpile.com/c/VKY8u6/JPA1) |
|  | BY4742 *cnb1Δ* | BY4742 *cnb1Δ*:: KAN | Deletion collection |
|  | YPL653 | MATa his3Δ0 leu2Δ0 ura3 Δ0 *tra1_Q3_*-HIS3 *tra1_Q3_*-HIS3  +DED1pr-YHR100-LEU2 *cnb1Δ*:: KAN | This study |
