# Supplementary material for: Evolutionary diversity of the control of the azole response by Tra1 across yeast species
Source: G3 (Bethesda). 2023 Oct 27;14(2):jkad250. doi: 10.1093/g3journal/jkad250 (PMC10849324; doi:10.1093/g3journal/jkad250)
Supplement: jkad250_Supplementary_Data [file jkad250_supplementary_data.zip › G3-2023-404639-T_Table_S2.docx]

**Table S2: Primers used in this study**

|  | **Primers** | | **Sequences** |
| --- | --- | --- | --- |
| *Candida albicans* | *CDR1* | Forward | 5' ACA AGA CCA GCA TCT CCA TAT AC 3' |
|  |  | Reverse | 5' ATC GAC GGA TCA CCT TTC ATA C 3' |
|  | *ERG11* | Forward | 5' GCT GCT GCC AAA GCT AAT TC 3' |
|  |  | Reverse | 5' TCT ATG TCT ACC ACC ACC AAA TG 3' |
|  | *ACT1* | Forward | 5' GTT GGT GAT GAA GCC CAA TCC 3' |
|  |  | Reverse | 5' CTG GAT GTT CTT CTG GAG CAA C 3′ |
| *Saccharomyces cerevisiae* | *PDR5* | Forward | 5’ CAA TGG CAA CGT ACG GTT TAT C 3’ |
|  |  | Reverse | 5’ GAT GGA GAC TTC AGC AA TGGA 3’ |
|  | *ERG11* | Forward | 5’ TGC ACC ATC CAT TGC ACT CT 3’ |
|  |  | Reverse | 5’ CCG ACG GAA TAA GAG GAG GC 3’ |
|  | *TDH3* | Forward | 5’ GTC CAC TCT TTG ACT GCT ACT C3’ |
|  |  | Reverse | 5’ TCG TAG GTG GTT TCC TTG TTC3’ |
