# Supplementary figures and images for: Evolutionary diversity of the control of the azole response by Tra1 across yeast species
Source: G3 (Bethesda). 2023 Oct 27;14(2):jkad250. doi: 10.1093/g3journal/jkad250 (PMC10849324; doi:10.1093/g3journal/jkad250)

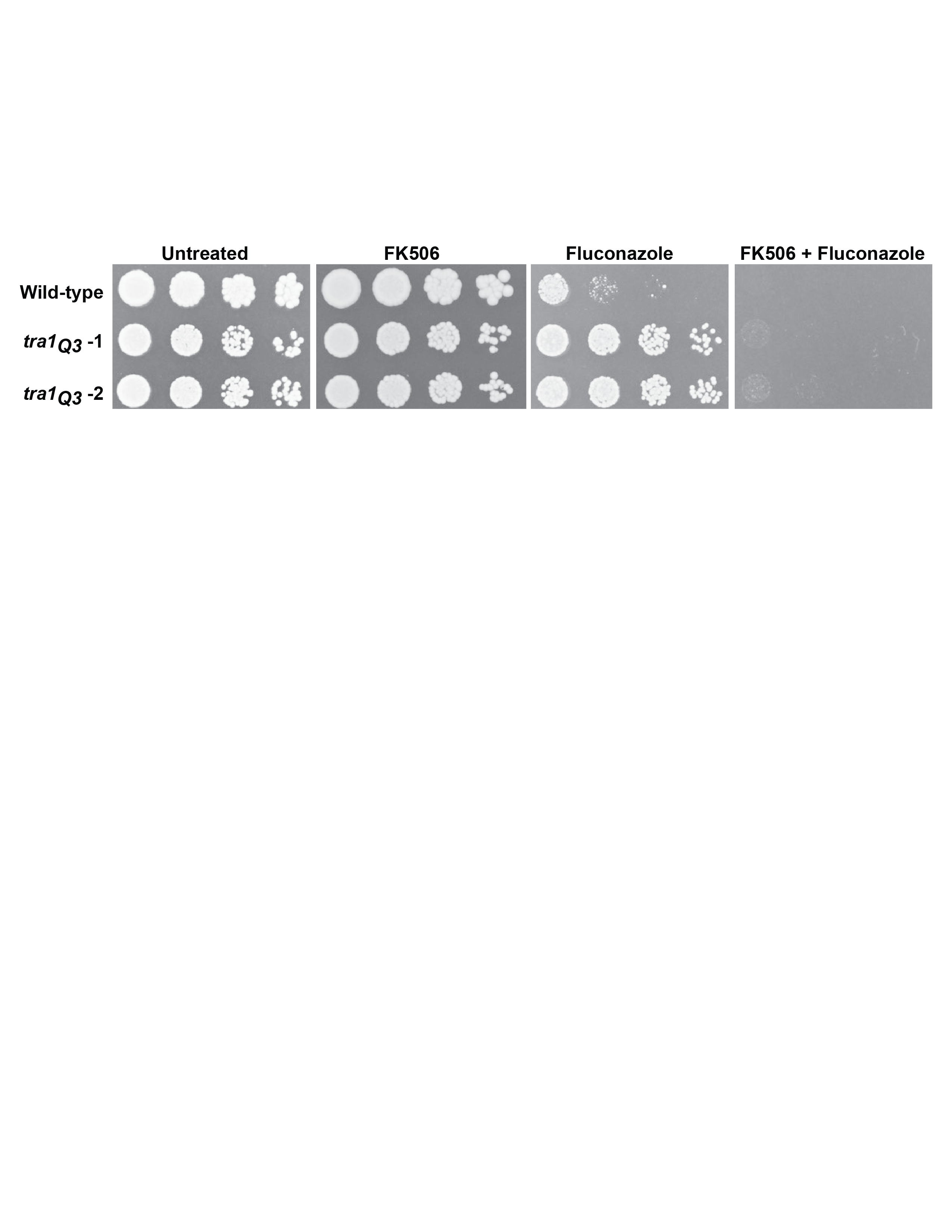

Supplement: jkad250_Supplementary_Data [file jkad250_supplementary_data.zip › G3-2023-404639-T_Figure_S1.png]

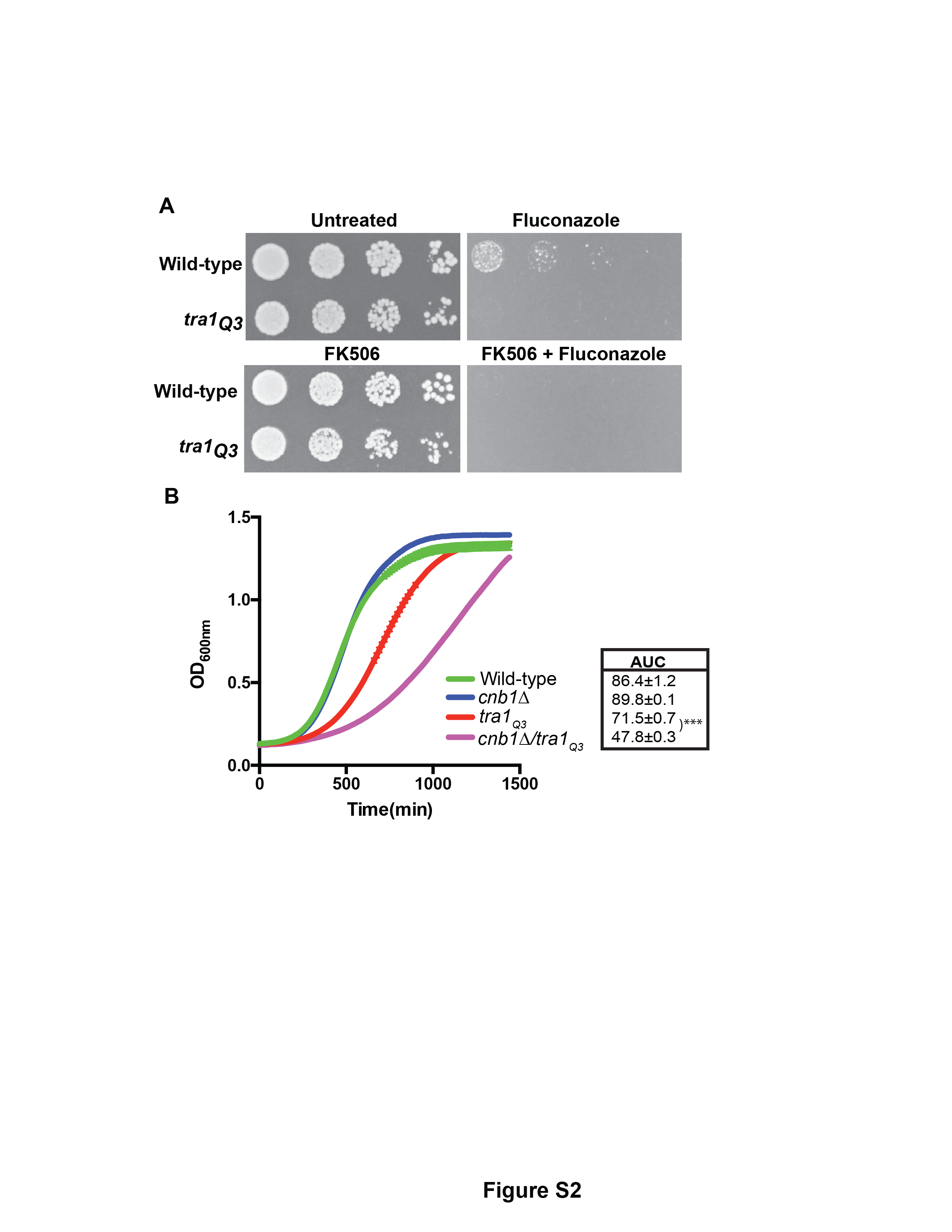

Supplement: jkad250_Supplementary_Data [file jkad250_supplementary_data.zip › G3-2023-404639-T_Figure_S2.png]
